# A cooperative control method and application for series multivariable coupled system

**DOI:** 10.1038/s41598-024-63169-7

**Published:** 2024-05-28

**Authors:** Yongchuan Yu, Haonan Yang, Shuo Wan, Qiusheng Liu, Jianzhuo Yan

**Affiliations:** 1 Faculty of Information Technology, Beijing University of Technology, Beijing, 100084 China; 2 Beijing South-to-North Water Diversion Project Tuancheng-Lake Management Office, Beijing, 100195 China

**Keywords:** Engineering, Mathematics and computing

## Abstract

Series multivariable coupled system is a typical controlled object in process control industry. The interaction of various state variables between multiple inputs and outputs in the system forms a complex series multivariable coupled structure. This coupled structure makes the control of a controlled object in the system affect the controlled object in the upper and lower control loop. As a result, it is difficult to control one or more control loops in the system without changing the state of other links in the system. In this paper, a cooperative control method for series multivariable coupled system is proposed. A decoupling controller is designed to remove the coupling effect caused by the interaction between stages, and the system is decoupled into several independent control loops. Differential leading PI (proportional-integral) error compensation method is introduced to ensure the following performance of the controller without static error. The proposed cooperative control method satisfies the Lyapunov stability, and has been successfully applied in the simulation experiment of cascade pumping station system of Beijing East-to-West water transfer project. The proposed method reduces the difficulty to controlling the water level of forebay of each pumping station and ensures the efficient operation of the cascade pumping station system.

## Introduction

**Figure 1:**
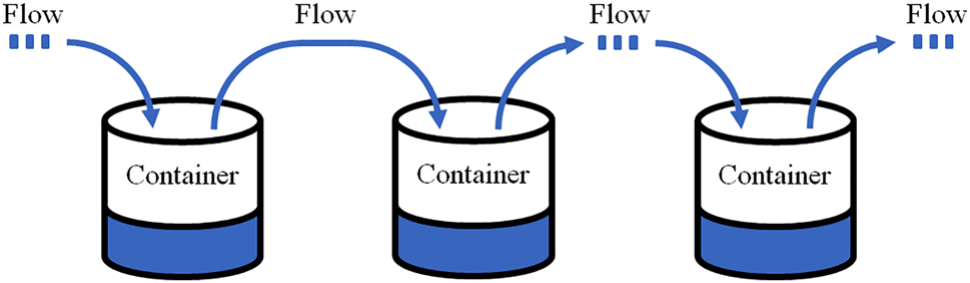
A series multivariable structure system.

In the modern control field, the control problem of complex controlled objects with multiple controlled links is generally described as a multivariable control system, one of which is shown in Fig. 1. The system consists of a cascade structure of multiple containers in series, and the liquid level of each container is controlled by controlling the flow between the containers.

The state variable of the above system is the liquid level of each container, and the control variable is the flow between containers. Since the flow between containers affects the liquid level state of the upper and lower levels at the same time, the state variables are coupled with each other, forming a series multivariable coupled system.

In this type of system, in the process of controlling any liquid level, it is necessary to change the flow of the upper and lower containers, which affects the state of the upper and lower containers, resulting in the flexible control of one or more states of the system becomes difficult^1^.

Series multivariable coupled systems are widely used in industrial production, such as cascade water transfer project^2–6^, multi-tank liquid level control system^7,8^, series reactor control for chemical production^9,10^, vehicle following model^11–13^ and power system^14–16^, which have strong typicality. If a type of control method is designed, it can realize the flexible cooperative control of the state variables of a certain link in the above application scenarios, which will have great practical value.

For series multivariable coupled system, the control of the system is complicated and difficult mainly because of the coupling relationship between the controlled objects. Therefore, how to solve the coupled problem between variables is the key to ensure the effective control of the system.

Traditional decoupling methods include the diagonal matrix method^17^, relative gain method, and characteristic curve method. However, when the order of the object transfer function and the dimensions of the input and output variables increase, the complexity of the controller designed by the traditional decoupling method increases greatly. The state feedback decoupling method fully utilizes the state information of the system and constructs a decoupling controller by designing a suitable state feedback matrix, thereby improving the control performance and stability of the system^18–20^. However, the implementation of the state feedback decoupling controller usually requires a lot of calculation, which may increase the computational overhead and implementation difficulty of the system. The inverse decoupling method^21–23^ uses the idea of feedback to ensure the realization of the decoupling controller, and the decoupling method has achieved remarkable results in practical applications^24–26^. However, the inverse decoupling method usually involves complex matrix operations, such as matrix inversion, eigenvalue decomposition, etc. These operations not only require a lot of computation, but also increase sharply with the increase of system scale. The simplified decoupling method^27^ requires that the transfer function matrix after decoupling is a diagonal transfer function matrix and does not impose any requirements on the diagonal part of the elements. The main advantage of simplified decoupling is that the calculation of the decoupling device is relatively simple. However, the elements of the decoupled diagonal transfer function are complicated. For the ideal decoupling method^28^, the transfer function matrix after decoupling must be very concise. Since the ideal decoupling method needs to invert the process transfer function matrix, when the order of the system is relatively high, the calculation of the decoupling device is very difficult. As one of the decoupling control methods, model predictive control has been widely used in industrial process control^29^, and has achieved good decoupling effect. The feedforward decoupling control method can fundamentally reduce or eliminate the coupling effect in a multivariable system by compensating for the nonlinear and time delay characteristics of the system in advance and can effectively resist the influence of external factors such as interference and parameter changes to achieve a better decoupling effect^30–32^.

Based on previous research, a cooperative control method for series multivariable coupled systems is proposed in this paper. The innovations of this paper are as follows:

1. The proposed decoupling controller can decouple the series multivariable coupled system, which not only realizes the whole cooperative control of the system, but also can control any one or more control loops without affecting the upper and lower control loops.
2. The proposed differential leading PI error compensation method can effectively suppress the influence of controller parameter error on control precision.
3. The proposed control method has been successfully applied in the cascade pumping station system of Beijing East-to-West Water Transfer Project, which significantly reduces the complexity of the system control and ensures the efficient and stable operation of the cascade pumping station system.

The remainder of this paper is structured as follows. Section "Problem description and system model" introduces the model construction of series multivariable coupled system. Section "Design and analysis of cooperative control methods" introduces the cooperative control method of series multivariable coupled system. Section "Application of cooperative control methods" applies the proposed cooperative control method to the simulation experiment of the cascade pumping station system in Beijing East-to-West Water Transfer Project and analyzes the experimental results. Section "Conclusion" presents the conclusion.

## Problem description and system model

As shown in Fig. 1, the state space equation of the series multivariable coupled system can be expressed as Eq. (1).

1 $$\left\{\begin{array}{c}{\dot{x}}_{1}(t)={{b}_{1}u}_{1}\left(t\right)+{{b}_{u,1}u}_{2}\left(t\right)+{{b}_{n,1}n}_{1}\left(t\right)\\ \\ {\dot{x}}_{2}(t)={{b}_{2}u}_{2}\left(t\right)+{{b}_{u,2}u}_{3}\left(t\right)+{{b}_{n,2}n}_{2}\left(t\right)\\ \\ \cdot \cdot \cdot \\ \\ {\dot{x}}_{n}(t)={{b}_{n}u}_{n}\left(t\right)+{{b}_{u,n}u}_{n+1}\left(t\right)+{{b}_{n,n}n}_{n}\left(t\right)\\ \\ {y}_{1}\left(t\right)={{c}_{1}x}_{1}\left(t\right), {y}_{2}\left(t\right)={{c}_{2}x}_{2}\left(t\right),\cdot \cdot \cdot ,{ y}_{n}(t)={{c}_{n}x}_{n}(t)\end{array}\right.$$

where $x$ represents the state variable, $y$ represents the system output, $u$ represents the control variable, $n$ represents the external disturbance, $b$ represents the input coefficient, ${b}_{u}$ represents the coupling coefficient, ${b}_{n}$ represents the disturbance coefficient, $c$ represents the output coefficient.

Then, the state space equation of the level $i$ control loop system of the system can be expressed as Eq. (2).

2 $$\left\{\begin{array}{c}{\dot{x}}_{i}\left(t\right)={{b}_{i}u}_{i}\left(t\right)+{{b}_{u,i}u}_{i+1}\left(t\right)+{{b}_{n,i}n}_{i}\left(t\right)\\ \\ {y}_{i}\left(t\right)={{c}_{i}x}_{i}\left(t\right)\end{array}\right.$$

**Figure 2:**
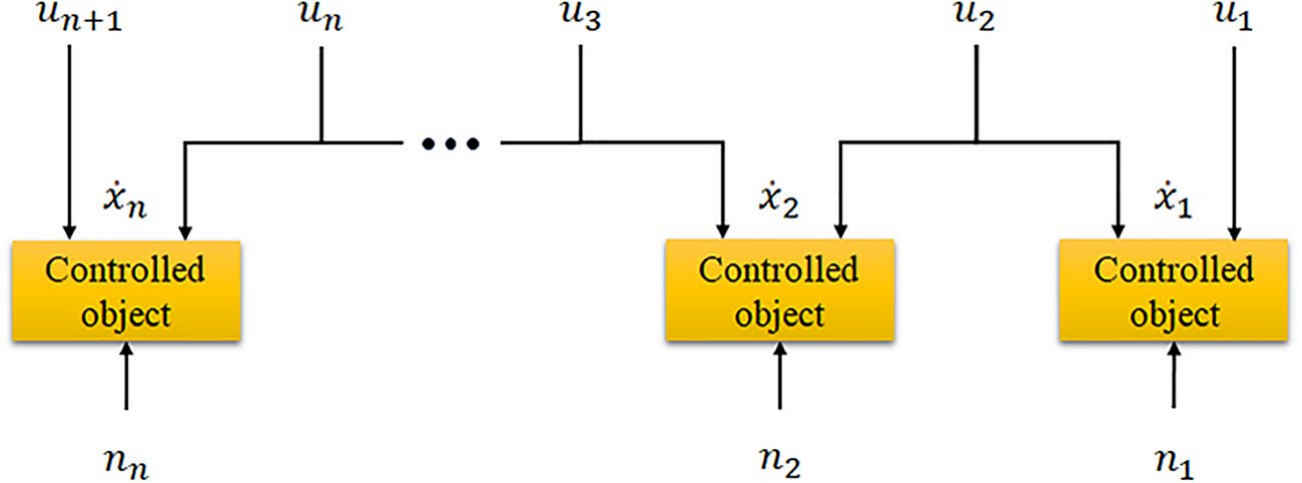
Schematic diagram of series multivariable coupled system.

The series multivariable coupled relationship makes the control variable of a certain control loop have a direct effect on the controlled object of this control loop, but also affect the controlled object of the upper control loop, which leads to a certain complexity of the control of a single or multiple controlled objects. The schematic diagram of series multivariable coupled system is shown in Fig. 2.

In order to effectively deal with the complex series multivariable coupled relationship in the system, it is necessary to design a special type of control method for decoupling the controlled objects in the system. In this way, it can ensure that the control of any one or more control loops is not affected by other loops, so as to achieve cooperative control of the system.

## Design and analysis of cooperative control methods

This section presents a cooperative control method for series multivariable coupled system. The main idea is to design a decoupling controller with double feedforward structure based on the control strategy of interstage feedback of control loops to decouple the system. On this basis, differential leading PI error compensation method is introduced to eliminate the problem of tracking error of given signal caused by lack of response of decoupling controller to parameter error.

### Methods comparison and analysis

In the process of decoupling control for a practical series multivariable coupled system, it is very important to find a simple, practical and easy-to-implement decoupling control method.

The decoupling control methods involving complex mathematical calculation and matrix solution, such as traditional decoupling method, state feedback decoupling method and inverse decoupling method, are often difficult to implement in practical engineering. These decoupling control methods are not suitable when facing a coupled system with large scale and many variables.

The model predictive control method needs to dynamically solve the optimization problem, which leads to a significant increase in computing time and cost, which limits the application of the method and is not suitable for practical engineering implementation.

Compared with the above classical decoupling control method, the feedforward control method does not need complicated mathematical calculation and model analysis, and is easy to implement in practical engineering. This characteristic makes the feedforward control method have significant advantages in dealing with series multivariable coupled engineering problems. Therefore, the cooperative control method proposed in this paper mainly uses the feedforward control method to design the decoupling controller.

### Decoupling controller

In order to speed up the response speed of the system, proportional control is introduced to the decoupling controller, and the input signal will first pass the proportional control, and the control variable obtained can be expressed as Eq. (3).

3 $${u}_{1,i}\left(t\right)={k}_{i}{r}_{i}\left(t\right),$$

where ${u}_{1,i}\left(t\right)$ represents the control variable of proportional control, ${k}_{i}$ represents the proportional coefficient, ${r}_{i}\left(t\right)$ represents the input signal.

For external disturbances, the disturbance will cause the output of the system to be biased. Therefore, the feedforward control is introduced to the decoupling controller to improve the control precision. After feedforward control, the control variable obtained can be expressed as Eq. (4).

4 $${u}_{2,i}\left(t\right)={k}_{i}{r}_{i}\left(t\right)+{g}_{1,i}\left(t\right){n}_{i}\left(t\right),$$

where ${u}_{2,i}\left(t\right)$ represents the control variable of feedforward control, ${g}_{1,i}\left(t\right)$ represents the feedforward response function.

For the series multivariable coupled problem of the system, the control variable of one control loop will affect the controlled object of the upper control loop, so the control variable of the control loop of this level can be regarded as the external disturbance to the upper control loop. To solve this problem, a feedforward control strategy based on interstage feedback of the control loop is proposed, and this control strategy is introduced to the decoupling controller. After interstage feedforward control, the control variable obtained can be expressed as Eq. (5).

5 $${u}_{i}\left(t\right)={k}_{i}{r}_{i}\left(t\right)+{g}_{1,i}\left(t\right){n}_{i}\left(t\right)+{g}_{2,i}\left(t\right){u}_{i+1}\left(t\right),$$

where ${u}_{i}\left(t\right)$ represents the control variable of interstage feedforward control, ${g}_{2,i}\left(t\right)$ represents the interstage feedforward response function.

To ensure that the feedforward control and interstage feedforward control fully compensate for the influence of the external disturbance and the control variable of the upper control loop, Eqs. (6) and (7) should be satisfied.

6 $${b}_{i}{g}_{1,i}\left(t\right)+{b}_{n,i}=0,$$

7 $${b}_{i}{g}_{2,i}\left(t\right)+{b}_{u,i}=0.$$

Then the feedforward response function and the interstage feedforward response function can be expressed as Eqs. (8) and (9), respectively.

8 $${g}_{1,i}\left(t\right)=-\frac{{b}_{n,i}}{{b}_{i}},$$

9 $${g}_{2,i}\left(t\right)=-\frac{{b}_{u,i}}{{b}_{i}}.$$

The control variable after the interstage feedforward control is the control variable of the decoupling controller, and the control variable finally acts on the controlled object. According to Eq. (3) to (9), the state variable of the system can be expressed as Eq. (10).

10 $${\dot{x}}_{i}\left(t\right)={b}_{i}{k}_{i}{r}_{i}\left(t\right).$$

Equation (10) shows that the controlled object of a certain control loop is not affected by the control variable of the next control loop, but is only related to the input signal, proportional coefficient and input coefficient of this control loop, which indicates that the decoupling controller decouples each control loop. The state space equation of the system is summarized as Eq. (11).

11 $$\left\{\begin{array}{c}{\dot{x}}_{i}\left(t\right)={b}_{i}{k}_{i}{r}_{i}\left(t\right)\\ \\ {y}_{i}\left(t\right)={{c}_{i}x}_{i}\left(t\right)\end{array}\right.$$

**Figure 3:**
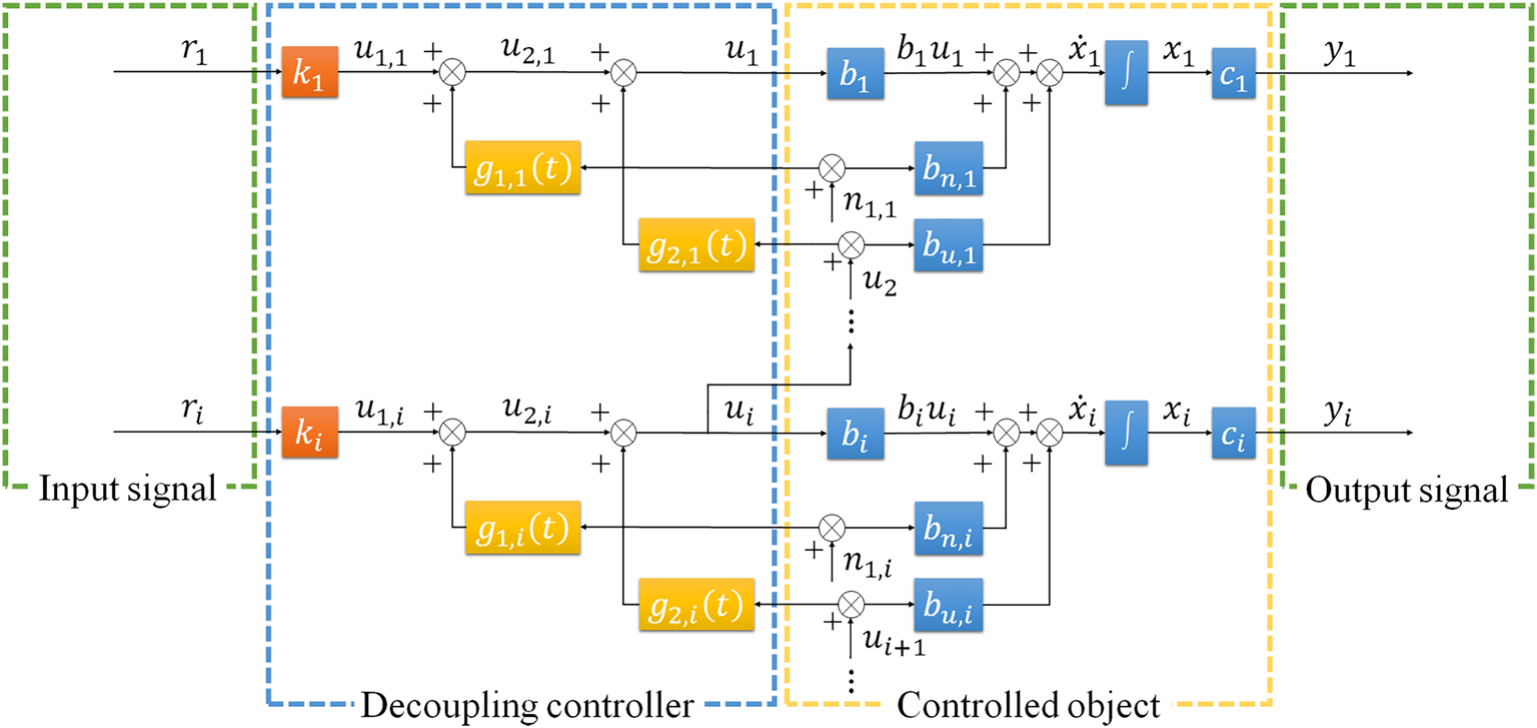
Schematic diagram of decoupling control system.

After the above design of the decoupling controller, the schematic diagram of decoupling control system is shown in Fig. 3.

### Error analysis of controller parameter

Considering that there is a certain amount of error in the proportional coefficient of the decoupling controller, the proportional coefficient with error can be expressed as Eq. (12).

12 $${k}_{\varepsilon ,i}={k}_{i}+{\varepsilon }_{1,i},$$

where ${k}_{\varepsilon ,i}$ represents the proportional coefficient with error, ${\varepsilon }_{1,i}$ represents the error of proportional coefficient.

Considering that the control variable also has a certain amount of error, the control variable with error can be expressed as Eq. (13).

13 $${u}_{\varepsilon ,i}\left(t\right)={u}_{i}\left(t\right)+{\varepsilon }_{2,i},$$

where ${u}_{\varepsilon ,i}(t)$ represents the control variable with error, ${\varepsilon }_{2,i}$ represents the error of control variable.

If the proportional coefficient and the control variable have errors, the output signal of the control system will also produce errors. The output signal at this time can be expressed as Eq. (14).

14 $${y}_{\varepsilon ,i}\left(t\right)={y}_{i}+{\int }_{0}^{t}{b}_{i}{c}_{i}\left({\varepsilon }_{1,i}{r}_{i}\left(t\right)+{\varepsilon }_{2,i}\right)dt,$$

where ${y}_{\varepsilon ,i}\left(t\right)$ represents the output signal with error.

Then the difference $d$ and ratio $p$ of the output signal with error and the output signal without error can be expressed as Eqs. (15) and (16), respectively.

15 $$d={\int }_{0}^{t}{b}_{i}{c}_{i}\left({\varepsilon }_{1,i}{r}_{i}\left(t\right)+{\varepsilon }_{2,i}\right)dt,$$

16 $$p=\frac{{y}_{\varepsilon ,i}}{{y}_{i}}.$$

Equations (15) and (16) show that the difference $d$ of the output signal with error and the output signal without error is mainly affected by the proportional coefficient and the error of the control variable, and will gradually increase or decrease with time. At the same time, the output signal with error will increase or decrease to $p$ times the output signal without error.

### Error compensation method

In order to suppress the negative influence of the decoupling controller parameter error on the output signal, it is necessary to introduce the error compensation method to the system to correct the controller error.

Obviously, the decoupling control system is an open-loop control system, and the output signal has no feedback loop to correct the error, so the robustness of the control system to the controller parameter error is poor, which affects the performance of the decoupling controller. Therefore, in order to realize the following performance of the decoupling controller to the given input signal without static error, the differential leading PI error compensation method is introduced on the basis of the original controller structure.

A closed-loop control system is formed by adding a negative feedback mechanism to the control system. At the same time, when the output signal is fed back, the differential leading strategy is adopted for the output signal. The differential leading is the differential variable of the system output signal fed back to the input signal. The system error can be expressed as Eq. (17).

17 $${e}_{i}(t)=r\left(t\right)-{y}{\prime}\left(t\right)$$

where ${e}_{i}(t)$ represents the system error, ${y}{\prime}\left(t\right)$ represents the differential variable of the system output.

PI control is introduced to the system error, and the control variable obtained can be expressed as Eq. (18).

18 $${u}_{0,i}\left(t\right)={k}_{P,i}{e}_{i}\left(t\right)+{k}_{I,i}{\int }_{0}^{t}{e}_{i}\left(t\right)dt,$$

where ${u}_{0,i}\left(t\right)$ represents the control variable of the PI control, ${k}_{P,i}$ represents the proportional coefficient of the PI control, ${k}_{I,i}$ represents the integral coefficient of the PI control.

The control variable after PI control is taken as the input variable of proportional control, and it can be further deduced that the state space equation of the system should be rewritten as Eq. (19).

19 $$\left\{\begin{array}{c}{\dot{x}}_{i}\left(t\right)={b}_{i}\left({k}_{\varepsilon , i}\left({k}_{P,i}{e}_{i}\left(t\right)+{k}_{I,i}{\int }_{0}^{t}{e}_{i}\left(t\right)dt\right)+{\varepsilon }_{2,i}\right)\\ \\ {y}_{i}\left(t\right)={{c}_{i}x}_{i}\left(t\right)\end{array}\right.$$

Equation (19) shows that the controlled object of a certain control loop is not affected by the control variable of the next control loop, but is only related to the input coefficient, the proportional coefficient of PI control, the integral coefficient of PI control, the proportional coefficient and the system error of this control loop, which indicates that the control system is still in a decoupling state under the error compensation method.

Under the action of the error compensation method, the output signal of the control system is corrected. The output signal at this time can be expressed as Eq. (20).

20 $${y}_{i}\left(t\right)={b}_{i}{c}_{i}{\int }_{0}^{t}{k}_{\varepsilon ,i}\left({k}_{P,i}{e}_{i}\left(t\right)+{k}_{I,i}{\int }_{0}^{t}{e}_{i}\left(t\right)dt\right)+{\varepsilon }_{2,i} dt.$$

**Figure 4:**
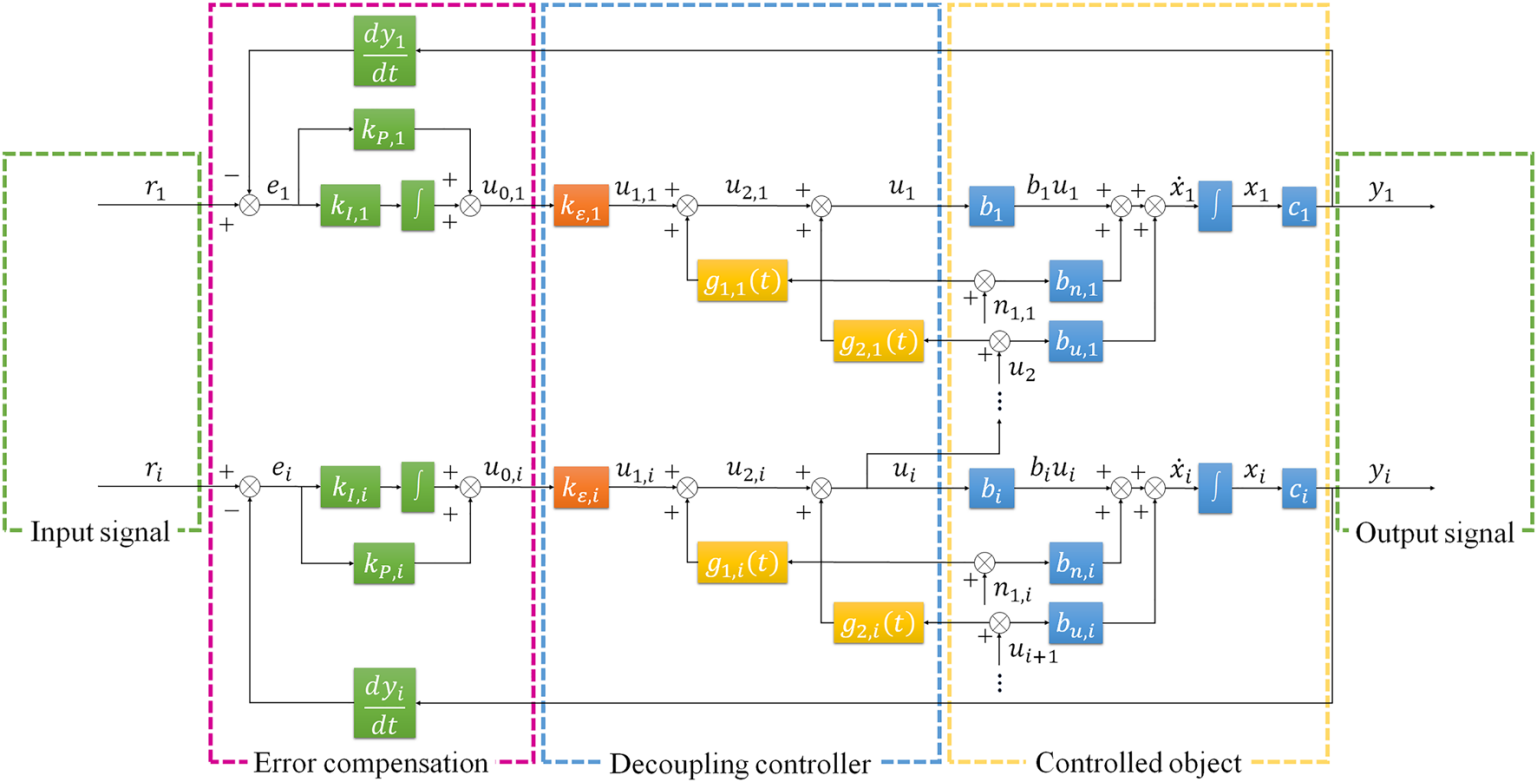
Schematic diagram of decoupling control system with error compensation.

Equation (20) shows that the error compensation method adds the proportional coefficient and integral coefficient of PI control. By adjusting these coefficients, the deviation caused by the parameter error of the decoupling controller to the output signal can be reduced, and the following performance of the controller to the given input signal without static error can be achieved, so that the controller has better adaptability. The schematic diagram of decoupling control system with error compensation is shown in Fig. 4.

### Stability analysis of control system

The decoupling control system is a closed-loop control system because the differential leading PI error compensation method introduces a negative feedback mechanism. In order to ensure the stability of the control system, it is necessary to conduct stability analysis of the control system. This section adopts the first method^33^ of Lyapunov stability theorem for analysis.

Equation (21) can be obtained from Eq. (17) and (19).

21 $${\dot{x}}_{i}\left(t\right)=-{b}_{i}{c}_{i}{k}_{\varepsilon ,i}{k}_{P,i}{x}_{i}\left(t\right)-{c}_{i}{k}_{\varepsilon ,i}{k}_{I,i}{\int }_{0}^{t}{x}_{i}\left(t\right) dt+{b}_{i}{k}_{\varepsilon ,i}{k}_{P,i}{r}_{i}\left(t\right)+{b}_{i}{k}_{\varepsilon ,i}{k}_{I,i}{\int }_{0}^{t}{r}_{i}\left(t\right) dt+{b}_{i}{\varepsilon }_{2,i}.$$

In the stability analysis of the control system, we only need to pay attention to the dynamic matrix related to the state variable and calculate the eigenvalue of the dynamic matrix, and can temporarily ignore the matrix unrelated to the state variable or output equation. Therefore, Eq. (21) can be simplified as Eq. (22).

22 $${\dot{x}}_{i}\left(t\right)=-{b}_{i}{c}_{i}{k}_{\varepsilon ,i}{k}_{P,i}{x}_{i}\left(t\right)-{c}_{i}{k}_{\varepsilon ,i}{k}_{I,i}{\int }_{0}^{t}{x}_{i}\left(t\right) dt.$$

Taking the derivative of ${x}_{i}$ with respect to time $t$ of Eq. (22) gives the Eq. (23).

23 $${\ddot{x}}_{i}\left(t\right)=-{b}_{i}{c}_{i}{k}_{\varepsilon ,i}{k}_{P,i}{\dot{x}}_{i}\left(t\right)-{c}_{i}{k}_{\varepsilon ,i}{k}_{I,i}{x}_{i}\left(t\right).$$

Obviously, Eq. (23) is a second-order differential equation. If we want to analyze the dynamic matrix of the system, we need to reduce the order of this equation. By introducing a new state variable ${z}_{i}\left(x,t\right)$, ${z}_{i}\left(x,t\right)$ can be expressed as the Eq. (24).

24 $${z}_{i}\left(x,t\right)=\left[\begin{array}{c}{x}_{i}\left(t\right)\\ {\dot{x}}_{i}\left(t\right)\end{array}\right].$$

Taking the derivative of ${x}_{i}$ with respect to time $t$ of Eq. (24) gives the Eq. (25).

25 $${\dot{z}}_{i}\left(x,t\right)=\left[\begin{array}{c}{\dot{x}}_{i}\left(t\right)\\ {\ddot{x}}_{i}\left(t\right)\end{array}\right].$$

From Eq. (23) to (25), Eq. (26) can be further derived.

26 $${\dot{z}}_{i}\left(x,t\right)=\left[\begin{array}{cc}0& 1\\ -{b}_{i}{c}_{i}{k}_{\varepsilon ,i}{k}_{P,i}& -{c}_{i}{k}_{\varepsilon ,i}{k}_{I,i}\end{array}\right]\left[\begin{array}{c}{x}_{i}\left(t\right)\\ {\dot{x}}_{i}\left(t\right)\end{array}\right].$$

For Eq. (26), Eq. (27) can be derived.

27 $${A}_{i}=\left[\begin{array}{cc}0& 1\\ -{b}_{i}{c}_{i}{k}_{\varepsilon ,i}{k}_{P,i}& -{c}_{i}{k}_{\varepsilon ,i}{k}_{I,i}\end{array}\right].$$

Then matrix ${A}_{i}$ is the dynamic matrix of the control system. The eigenvalue of the dynamic matrix can be obtained by calculating the characteristic polynomial of this dynamic matrix, which can be expressed as Eq. (28).

28 $$det\left({A}_{i}-{\lambda }_{i}I\right)=0,$$

where ${\lambda }_{i}$ represents the eigenvalue to be calculated, $I$ represents the identity matrix of the same order as ${A}_{i}$.

Equation (28) can be expanded to get Eq. (29).

29 $${{\lambda }_{i}}^{2}+{c}_{i}{k}_{\varepsilon ,i}{k}_{I,i}{\lambda }_{i}+{b}_{i}{c}_{i}{k}_{\varepsilon ,i}{k}_{P,i}=0.$$

According to the derivation formula, the eigenvalue can be calculated, and its roots can be expressed as Eqs. (30) and (31).

30 $${\lambda }_{i,1}=\frac{-{c}_{i}{k}_{\varepsilon ,i}{k}_{I,i}+\sqrt{{c}_{i}^{2}{k}_{\varepsilon ,i}^{2}{k}_{I,i}^{2}-4{b}_{i}{c}_{i}{k}_{\varepsilon ,i}{k}_{P,i}}}{2{c}_{i}{k}_{\varepsilon ,i}{k}_{I,i}},$$

31 $${\lambda }_{i,2}=\frac{-{c}_{i}{k}_{\varepsilon ,i}{k}_{I,i}-\sqrt{{c}_{i}^{2}{k}_{\varepsilon ,i}^{2}{k}_{I,i}^{2}-4{b}_{i}{c}_{i}{k}_{\varepsilon ,i}{k}_{P,i}}}{2{c}_{i}{k}_{\varepsilon ,i}{k}_{I,i}}.$$

Equations (30) and (31) show that the eigenvalues are closely related to each parameter of the control system. Therefore, in the process of subsequent simulation experiments, it is necessary to analyze and reasonably set the value range of each parameter according to the actual situation, so that the eigenvalues ${\lambda }_{i,1}$ and ${\lambda }_{i,2}$ are located in the left half of the complex plane, so that the control system has good stability.

## Application of cooperative control methods

In order to verify the effectiveness of the cooperative control method, the proposed method is applied to the cascade pumping station system of Beijing East-to-West Water Transfer Project for simulation experiments and analysis.

### Overview of project

**Figure 5:**
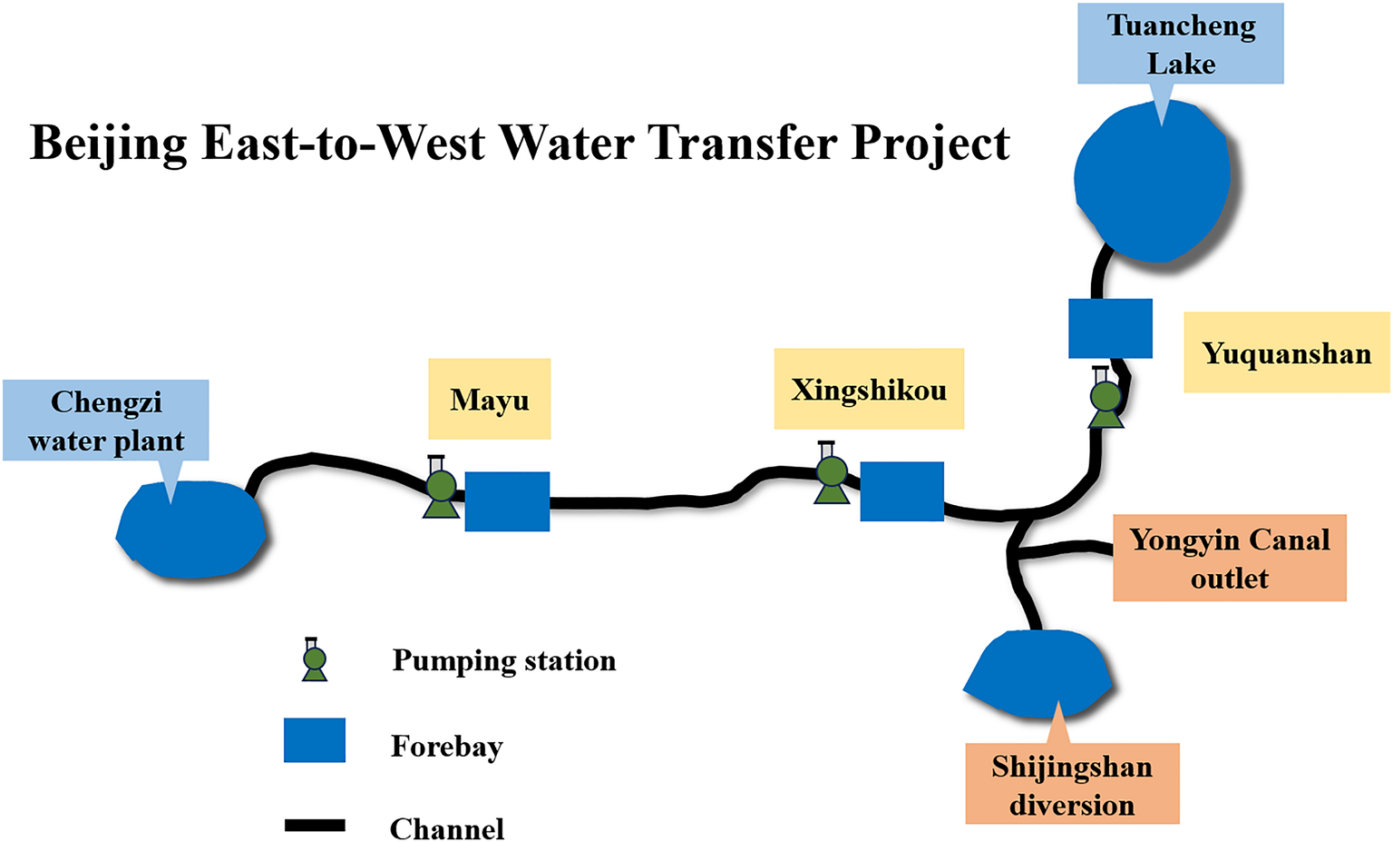
Layout diagram of Beijing East-to-West water transfer project.

Beijing East-to-West Water Transfer Project is an important part of the Beijing water transfer project, its main purpose is to supply water to Mentougou Chengzi water plant. The project uses pipelines to carry water and sets up a 3-stage pumping station. The water transfer route starts from Tuancheng Lake in Guhe Garden in the east and ends at Mentougou Chengzi water plant in the west, passing through three pumping stations including Yuquanshan pumping station, Xingshikou pumping station and Mayu pumping station. The water plant will adjust the demand flow several times a day, and the output flow of the Mayu pumping station needs to meet the water demand of the water plant. In East-to-West Water Transfer Project, Yuquanshan forebay is connected with Tuancheng Lake water intake, the water level of Yuquanshan forebay is not controlled, only the water level of Xingshikou forebay and Mayu forebay can be controlled. Between Yuquanshan pumping station and Xingshikou pumping station, there are Shijingshan diversion and Yongyin Canal outlet. During the operation of the project, the water level of the forebay must be kept within the range of the scheduling water level. It can be seen that this project can be regarded as a cascade pumping station system. The layout diagram of Beijing East-to-West Water Transfer Project is shown in Fig. 5. The relevant parameter information of each pumping station is shown in Table 1. The relevant information of the water level of each forebay is shown in Table 2.

**Table 1:** Relevant parameter information of each pumping station.

Number of units in pumping stations	Maximum operating flow (m^3^/h)	Minimum operating flow (m^3^/h)
3 large units in Yuquanshan	9200	5800
3 small units in Yuquanshan	5400	5400
4 units in Xingshikou	2100	2100
3 units in Mayu	2100	2100

**Table 2:** Relevant information of water level of each forebay.

Forebay	Cross-sectional area (m^2^)	Minimum water level (m)	Maximum water level (m)	Safe water level (m)	The range of the scheduled water level (m)
Xingshikou	1800	62.5	65.0	64.65	64.5–64.9
Mayu	650	92.5	95.0	94.7	94.5–95.0

**Figure 6:**
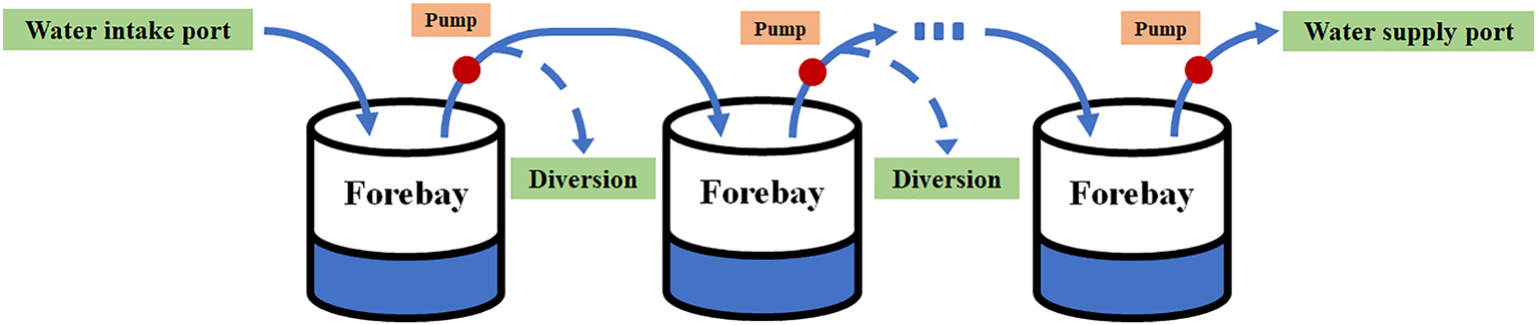
Structural diagram of the cascade pumping station.

The cascade pumping station system is similar to the series multivariable structure system in Fig. 1, and its structure diagram is shown in Fig. 6.

The cascade pumping station system is connected with the forebay through multiple pumping stations. Water is drawn from the intake port, and the flow is controlled by the pumping station until it reaches the water supply port. It should be noted that there may be a diversion between the pumping stations, which will cause some water loss. The flow of the last stage pumping station is carried out according to the water demand of the water supply outlet. Therefore, the cascade pumping station system of Beijing East-to-West Water Transfer Project can be transformed into a series multivariable coupled system.

### Parameters analysis of project

In order to better understand the actual physical meaning of each parameter of the control system in East-to-West Water Transfer Project and facilitate the parameter setting of the subsequent simulation experiment, these parameters will be analyzed in detail in this section.

In the decoupling control system, the input signal is the expected adjustment speed of the water level of forebay of each pumping station, the control variable is the output flow of each pumping station, the controlled object is the water level of each forebay, and the output signal is the current water level of each forebay. The diversion flow results in a decrease in the amount of water flowing into the forebay and is therefore considered an external disturbance.

The actual adjustment speed of the water level of each forebay is related to the pumping station, the diversion, the output flow of the next pumping station and the forebay area, and its expression can be expressed as Eq. (32).

32 $${V}_{i}\left(t\right)=\frac{{Q}_{i}\left(t\right)-{Q}_{i+1}\left(t\right)-{Q}_{D,i}\left(t\right)}{{S}_{i}},$$

where ${V}_{i}\left(t\right)$ represents the actual adjustment speed of the water level of each forebay, ${Q}_{i}\left(t\right)$ represents the output flow of each pumping station, ${Q}_{D,i}\left(t\right)$ represents the diversion flow, ${S}_{i}$ represents the forebay area.

The control variable of the control system is the output flow of each pumping station, and the output signal is the current water level of each forebay. Combined with Eq. (32), it can be seen that the input coefficient of the system is the reciprocal of the area of each forebay, the coupling coefficient and disturbance coefficient are the negative reciprocal of the area of each forebay, and the first derivative of the state variable is the actual adjusting speed of the water level of each forebay. Its expression can be expressed as Eqs. (33) to (35).

33 $${b}_{i}=\frac{1}{{S}_{i}},$$

34 $${b}_{u,i}={b}_{n,i}=-\frac{1}{{S}_{i}},$$

35 $${V}_{i}\left(t\right)=\frac{{k}_{i}{V}_{E,i}\left(t\right)}{{S}_{i}},$$

where ${V}_{E,i}\left(t\right)$ represents the expected adjustment speed of the water level of each forebay.

From Eqs. (8), (9), (33) and (34), the feedforward response function and the interstage feedforward response function can be derived as Eqs. (36) and (37).

36 $${g}_{1,i}\left(t\right)=1,$$

37 $${g}_{2,i}\left(t\right)=1.$$

According to Eq. (32), the expression of output flow of each pumping station can be expressed as Eq. (38).

38 $${Q}_{i}\left(t\right)={S}_{i}{V}_{i}\left(t\right)+{Q}_{i+1}\left(t\right)+{Q}_{D,i}\left(t\right).$$

From Eqs. (5), (36) to (38), it can be seen that the proportional coefficient is the area of each forebay, and the error of the forebay area will lead to the error of the proportional coefficient. At the same time, the flow error of pumping station is caused by the characteristic error of the unit. These two types of project parameter errors will eventually affect the control accuracy of forebay water level change rate.

In East-to-West water transfer project, the output flow of Mayu pumping station is adjusted according to the water demand of Chengzi water plant, and it needs to meet the water demand of the water plant. Therefore, the control variable ${u}_{n+1}$ in Eq. (1) is equivalent to the water demand of the water plant.

In the control system, the state variable ${x}_{i}$ is the current water level of each forebay, and the output signal is also the current water level of each forebay. Therefore, the output coefficient ${c}_{i}$ of the state space equation can be expressed as Eq. (39).

39 $${c}_{i}=1.$$

### Simulation experiment of decoupling control system

In order to verify the effectiveness of the decoupling controller in practical project. This section applies the decoupling control system to the simulation experiment of East-to-West water transfer project, and analyzes the experiment results.

#### Parameter setting of project

It is assumed that the flow of Shijingshan diversion is 3000 m^3^/h. The flow of Yongyin Canal outlet is usually 0, so it is not considered in the simulation experiment. The output flow of Mayu pumping station needs to meet the demand flow of the water plant. It is assumed that the water demand of Chengzi water plant is 3000 m^3^/h, so the control variable ${u}_{3}$ is 3000. The areas of Xingshikou forebay and Mayu forebay are 1800 m^2^ and 650 m^2^ respectively. The parameters of the decoupling control system in East-to-West Water Transfer Project are shown in Table 3.

**Table 3:** Parameter setting of decoupling control system in East-to-West water transfer project.

Description	Parameter	Value	Description	Parameter	Value
Proportional coefficient	${k}_{1}$	1800	Coupling coefficient	${b}_{u,1}$	− 1/1800
	${k}_{2}$	650		${b}_{u,2}$	− 1/650
Input coefficient	${b}_{1}$	1/1800	Output coefficient	${c}_{1}$	1
	${b}_{2}$	1/650		${c}_{2}$	1
Disturbance coefficient	${b}_{n,1}$	− 1/1800	External disturbance	${n}_{1}$	3000
	${b}_{n,2}$	− 1/650		${n}_{2}$	0

#### Results and analysis of project simulation experiment

This section uses MATLAB Simulink simulation environment to carry out the project simulation experiment of the decoupling control system. As the complex environment and changeable conditions in actual project have very high requirements on the control system, three possible practical conditions are considered in this section. The detailed descriptions of each practical condition are shown in Table 4.

**Table 4:** Descriptions of conditions in East-to-West water transfer project.

Condition	Description
1	Only adjust one forebay
2	Adjust one forebay first, and then adjust the other forebay
3	Adjust Xingshikou and Mayu forebay at the same time

**Figure 7:**
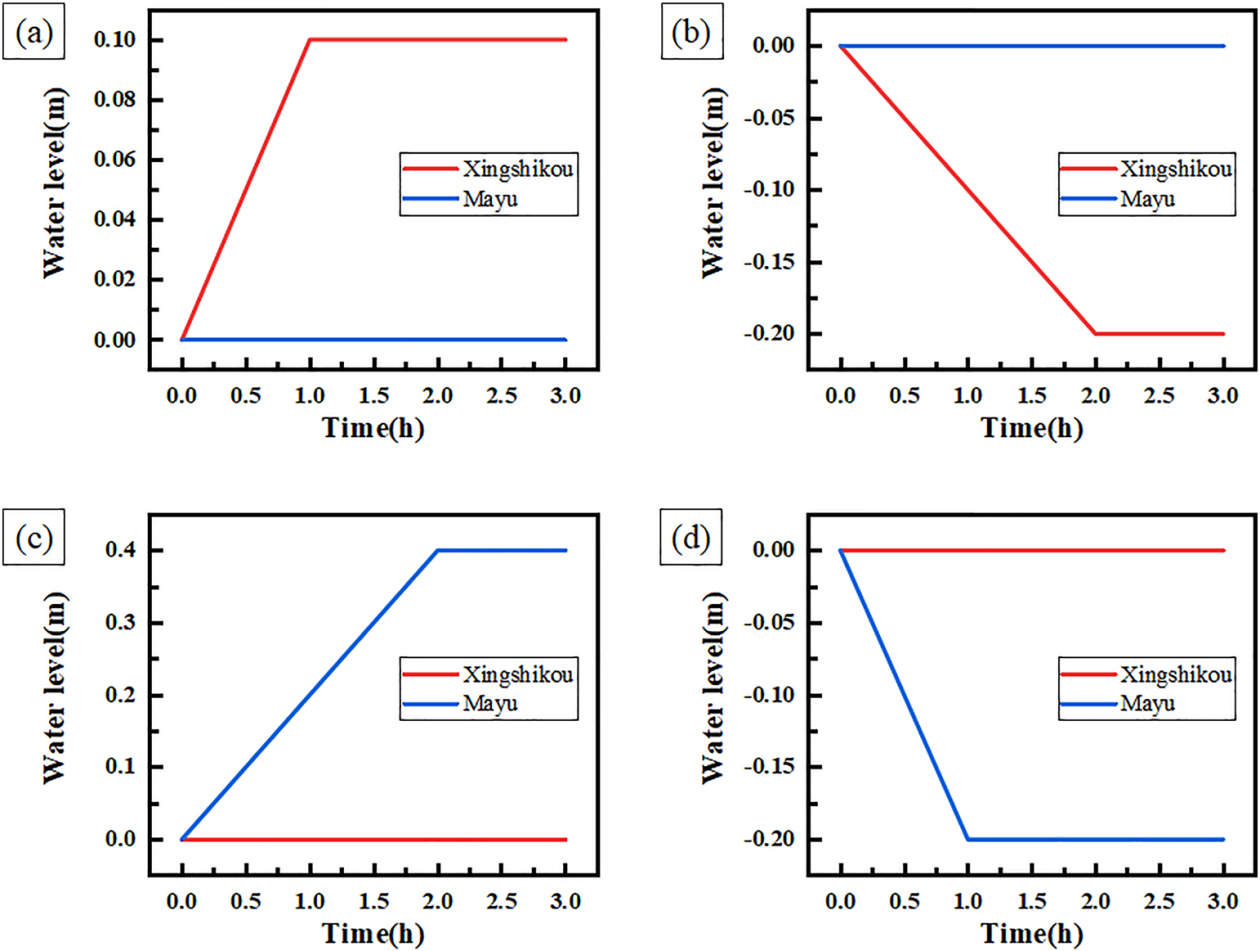
Curves of water level change of condition 1.

Through simulation experiments, the experiment results and the curves of water level change of condition 1 are shown in Table 5 and Fig. 7 respectively.

**Table 5:** Experiment results of condition 1.

Experiment number	Forebay	Control time (h)	Expected adjustment speed (m/h)	Theoretical water level change (m)	Actual water level change (m)
1	Xingshikou	1	0.1	0.1	0.1
	Mayu	0	0	0	0
2	Xingshikou	2	− 0.1	− 0.2	− 0.2
	Mayu	0	0	0	0
3	Xingshikou	0	0	0	0
	Mayu	2	0.2	0.4	0.4
4	Xingshikou	0	0	0	0
	Mayu	2	− 0.1	− 0.2	− 0.2

Table 5 shows that the actual water level change of Xingshikou and Mayu forebay is equal to the theoretical water level change, which indicates that the water level of the forebay is adjusted according to the expected adjustment speed and control time. Figure 7 shows that when the decoupling controller controls the water level of Xingshikou forebay, the water level of Mayu forebay remains stable. After the control of the water level of Xingshikou forebay, the water level of Xingshikou forebay and Mayu forebay remains stable. When the decoupling controller controls the water level of Mayu forebay, the water level of Xingshikou forebay remains stable, and the water level of Xingshikou forebay and Mayu forebay remains stable after the end of the control.

This shows that when the decoupling controller controls a single water level of forebay, it does not affect water levels of other forebays. The simulation experiment results verify the decoupling of the decoupling controller.

**Figure 8:**
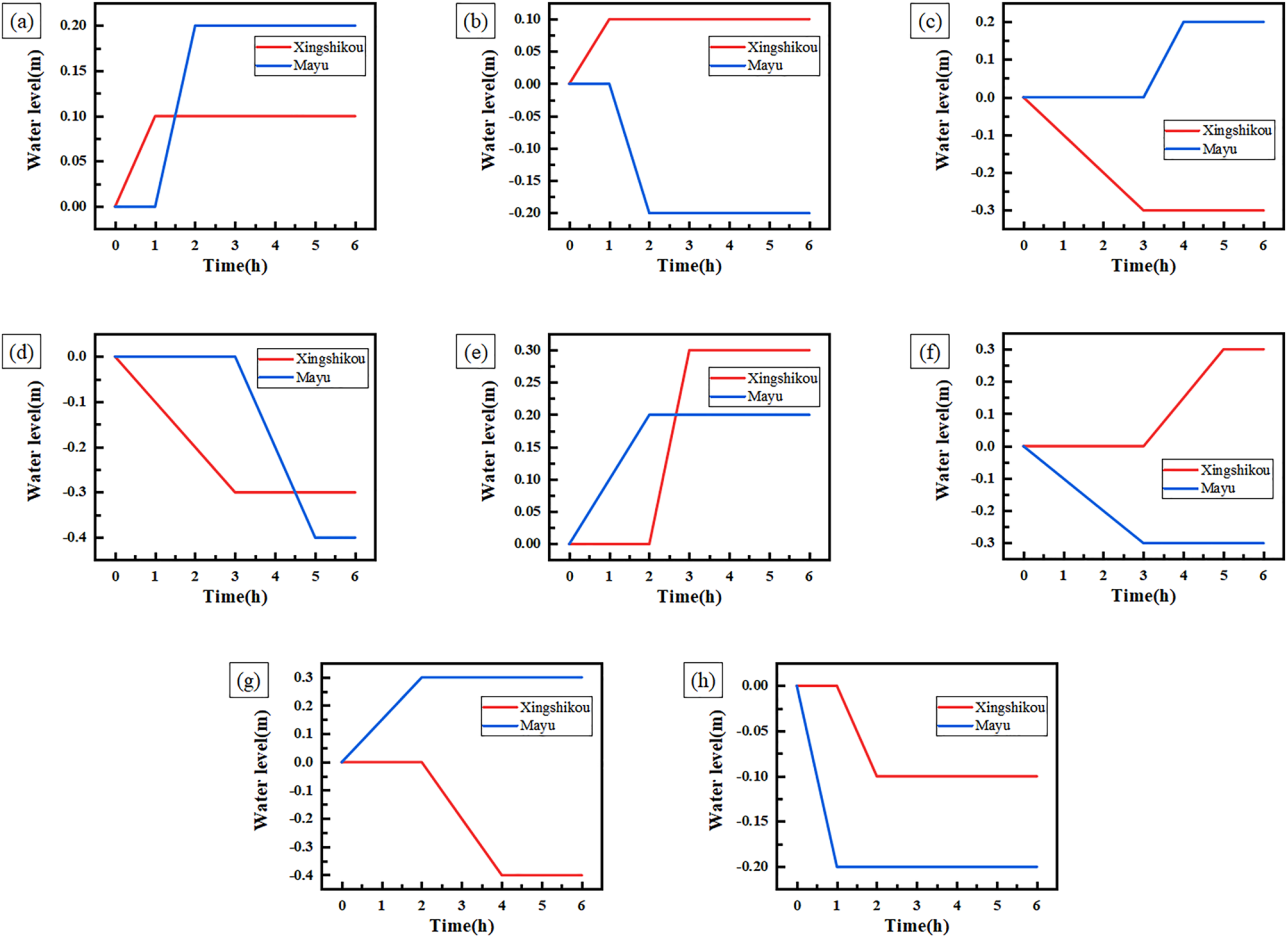
Curves of water level change of condition 2.

The experiment results and the curves of water level change of condition 2 are shown in Table 6 and Fig. 8 respectively.

**Table 6:** Experiment results of condition 2.

Experiment number	Forebay	Control time (h)	Expected adjustment speed (m/h)	Theoretical water level change (m)	Actual water level change (m)
1	Xingshikou	1	0.1	0.1	0.1
	Mayu	1	0.2	0.2	0.2
2	Xingshikou	1	0.1	0.1	0.1
	Mayu	1	− 0.2	− 0.2	− 0.2
3	Xingshikou	3	− 0.1	− 0.3	− 0.3
	Mayu	1	0.2	0.2	0.2
4	Xingshikou	3	− 0.1	− 0.3	− 0.3
	Mayu	2	− 0.2	− 0.4	− 0.4
5	Xingshikou	1	0.3	0.3	0.3
	Mayu	2	0.1	0.2	0.2
6	Xingshikou	2	0.15	0.3	0.3
	Mayu	3	− 0.1	− 0.3	− 0.3
7	Xingshikou	2	− 0.2	− 0.4	− 0.4
	Mayu	2	0.15	0.3	0.3
8	Xingshikou	1	− 0.1	− 0.1	− 0.1
	Mayu	1	− 0.2	− 0.2	− 0.2

Table 6 shows that the actual water level change of Xingshikou and Mayu forebay is equal to the theoretical water level change, which indicates that the water level of the forebay is adjusted according to the expected adjustment speed and control time. Figure 8 shows that in the experiments of groups 1 to 4 (Fig. 8a–d), the decoupling controller first controls the water level of Xingshikou forebay. During the control process, the water level of Mayu forebay remains stable. After the control of Xingshikou forebay is completed, the decoupling controller controls the water level of Mayu forebay, while the water level of of Xingshikou forebay remains stable. After the control of Mayu forebay is completed, the water levels of Xingshikou forebay and Mayu forebay remain stable. In the experiments of group 5 to 8 (Fig. 8e–h), the decoupling controller first controls the water level of Mayu forebay. During the control process, the water level of Xingshikou forebay remains stable. After the control of Mayu forebay is completed, the decoupling controller controls the water level of Xingshikou forebay, while the water level of of Mayu forebay remains stable. After the control of Xingshikou forebay is completed, the water levels of Xingshikou forebay and Mayu forebay remain stable.

This shows that the decoupling controller can not only control the water level of Xingshikou forebay or Mayu forebay, but also can control water level of one forebay first and then control water level of another forebay. The simulation experiment results not only verify the decoupling of the decoupling controller, but also show that the decoupling controller has the flexibility to control the water level of each forebay.

**Figure 9:**
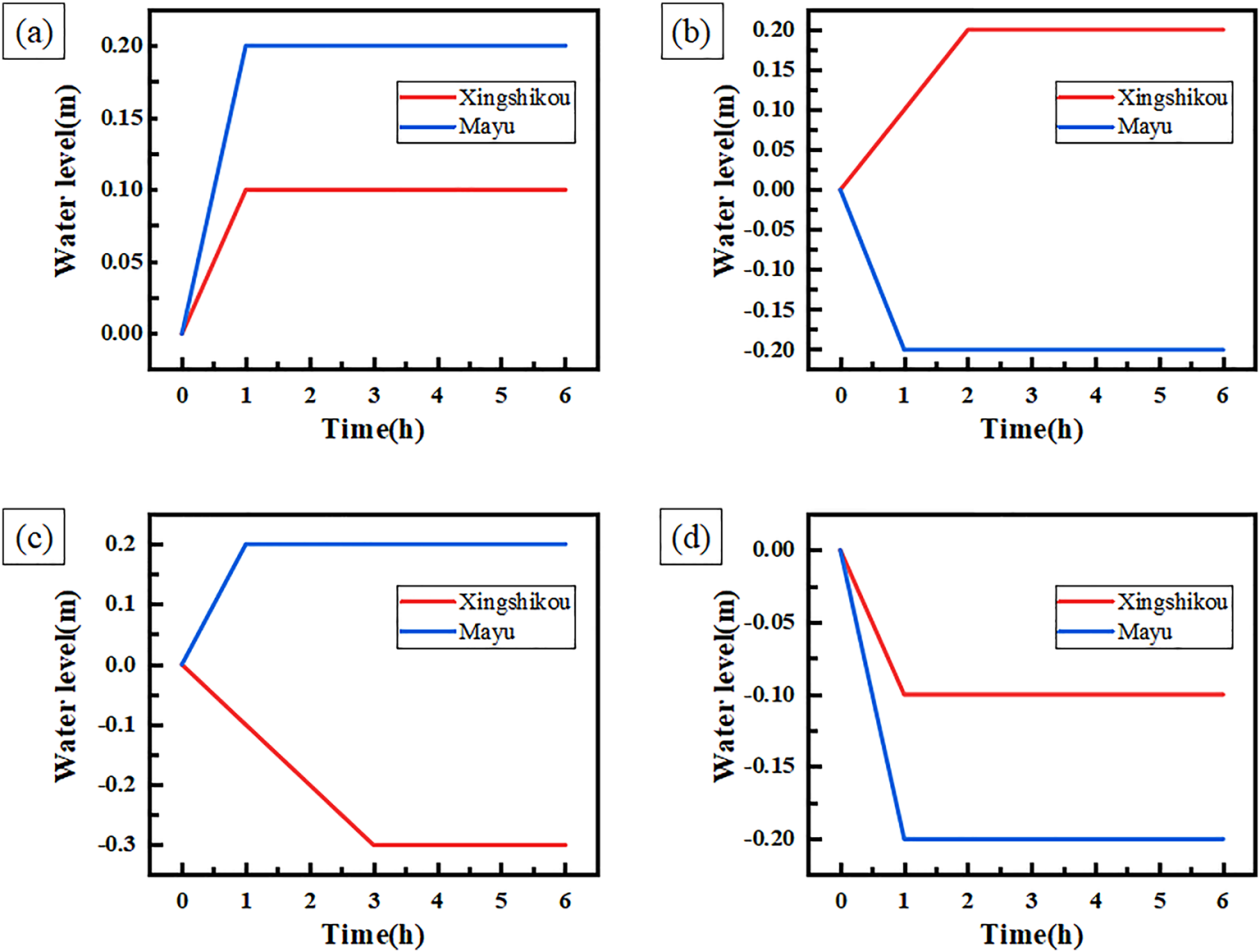
Curves of water level change of condition 3.

The experiment results and the curves of water level change of condition 3 are shown in Table 7 and Fig. 9 respectively.

**Table 7:** Experiment results of condition 3.

Experiment number	Forebay	Control time (h)	Expected adjustment speed (m/h)	Theoretical water level change (m)	Actual water level change (m)
1	Xingshikou	1	0.1	0.1	0.1
	Mayu	1	0.2	0.2	0.2
2	Xingshikou	2	0.1	0.2	0.2
	Mayu	1	− 0.2	− 0.2	− 0.2
3	Xingshikou	3	− 0.1	− 0.3	− 0.3
	Mayu	1	0.2	0.2	0.2
4	Xingshikou	1	− 0.1	− 0.1	− 0.1
	Mayu	1	− 0.2	− 0.2	− 0.2

Table 7 shows that the actual water level change of Xingshikou and Mayu forebay is equal to the theoretical water level change, which indicates that the water level of the forebay is adjusted according to the expected adjustment speed and control time. Figure 9 shows that the decoupling controller can control the water level of Xingshikou forebay and Mayu forebay at the same time, and the water level can remain stable after the end of the water level control of the forebay.

In fact, the decoupling of the decoupling controller makes the system already have the ability to control the water level of one forebay, and the simultaneous control of the water level of Xingshikou forebay and Mayu forebay can be regarded as the separate control of the water level of Xingshikou forebay and Mayu forebay. The simulation experiment results not only verify the decoupling ability of the decoupling controller, but also show that the controller can control the water level of each forebay at the same time.

### Simulation experiment of decoupling control system with error compensation

In order to fully verify the performance of differential leading PI error compensation method to follow the given input signal without static error in the simulation experiment of East-to-West Water Transfer Project. This section analyzes the error of the parameters of the East-to-West Water Transfer Project, and applies the decoupling control system with error compensation method to the simulation experiment of East-to-West Water Transfer Project, and analyzes the experiment results.

#### Error analysis of project parameter

In the cascade pumping station system, the control variable of the decoupling controller is the flow of each pumping station, and the characteristic error of pumping station units will cause some error for the flow. At the same time, the proportional coefficient of the decoupling controller is the area of each forebay, and the area measured often has a certain error. According to the actual situation of East-to-West Water Transfer Project, considering the error of the flow is within the range of ± 5%, and the error of the forebay area is within the range of ± 2%.

This section conducts simulation experiments on the water levels of Xingshikou forebay and Mayu forebay respectively according to the above error parameters, and calculates the error rate $\vartheta$ of the output signal with error and the output signal without error. The expression of error rate can be expressed as Eq. (40).

40 $$\vartheta =\frac{{y}_{\varepsilon ,i}-{y}_{i}}{{y}_{i}}.$$

**Figure 10:**
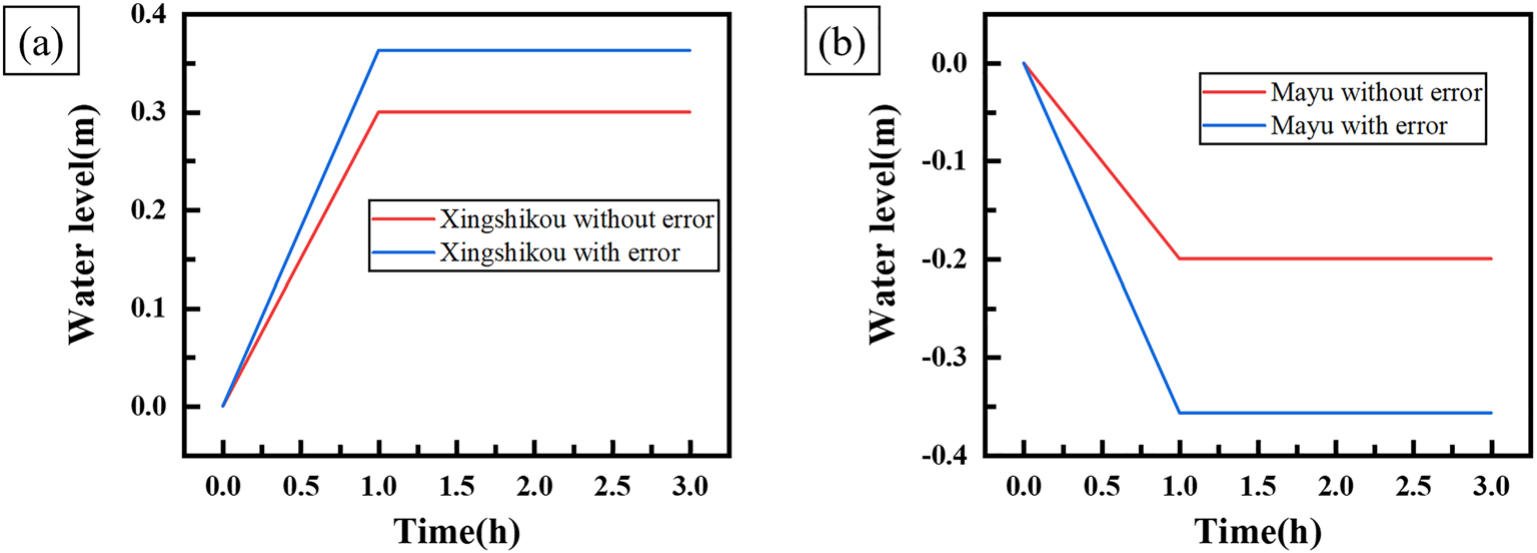
Curves of water level change of each forebay with error.

Through simulation experiments, the experiment results and the curves of water level change of each forebay are shown in Table 8 and Fig. 10 respectively.

**Table 8:** Results of water level change of each forebay with error.

Forebay	Control time (h)	Expected adjustment speed (m/h)	Theoretical water level change (m)	Actual water level change (m)	Error (m)	Error rate (%)
Xingshikou	1	0.3	0.3	0.363	0.063	21
Mayu	1	− 0.2	− 0.2	− 0.357	− 0.157	78.5

Table 8 and Fig. 10 show that when the decoupling controller controls the water level of Xingshikou forebay, the expected adjustment speed is 0.3 m/h and the control time is 1 h. After the end of the water level control, the theoretical change of water level of Xingshikou forebay should be 0.3 m, but the actual change of water level is 0.363 m, which is 0.063 m higher than the theoretical value, and the error rate is 21%. When the decoupling controller controls the water level of Mayu forebay, the expected adjustment speed is -0.2 m/h and the control time is 1 h. After the end of the water level control, the theoretical water level change should be − 0.2 m, but the actual water level change is − 0.357 m, which is 0.157 m lower than the theoretical value, and the error rate is 78.5%.

The simulation experiment results show that the characteristic error of units and the error of the forebay area will have a great influence on the control accuracy of the water level change rate of Xingshikou forebay and Mayu forebay.

#### Results and analysis of project simulation experiment

In order to improve the control accuracy of the decoupling controller on the change rate of the water level of each forebay and realize the performance of the decoupling controller on the given input signal without static error. This section uses the decoupling controller with error compensation method to carry out simulation experiments to verify the effectiveness of the error compensation method. Table 9 lists the value ranges and settings of parameters for the error compensation method. According to the parameters, the calculated eigenvalues are located in the left half of the complex plane, which fully shows that the control system has good stability.

**Table 9:** Parameters of error compensation method.

Parameter	Value range	Value
${k}_{P,1}$	[0, 1]	0.5
${k}_{I,1}$	[0, 100]	80
${k}_{P,2}$	[0, 1]	0.5
${k}_{I,2}$	[0, 100]	80

**Figure 11:**
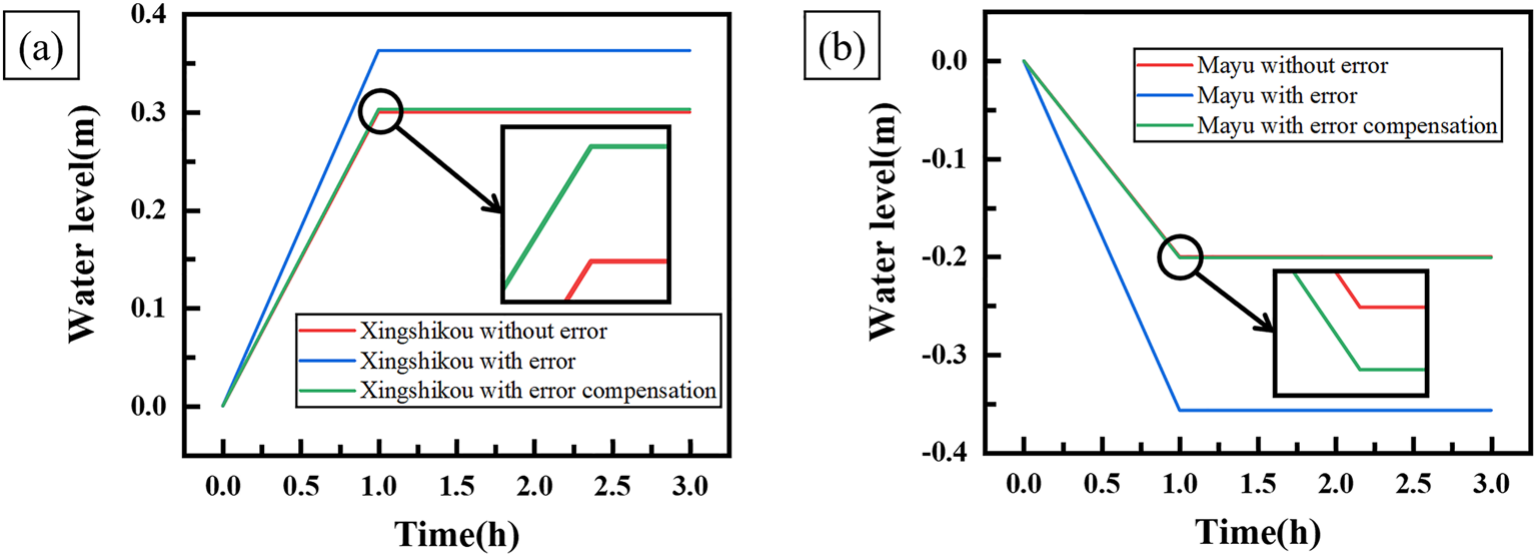
Curves of water level change of each forebay with error compensation.

Through simulation experiments, the experiment results and the curves of water level change of each forebay are shown in Table 10 and Fig. 11 respectively.

**Table 10:** Results of water level change of each forebay with error compensation.

Forebay	Theoretical water level change (m)	Actual water level change (m)	Error (m)	Error rate (%)
Xingshikou	0.3	0.303	0.003	1
Mayu	− 0.2	− 0.201	− 0.001	0.5

Tables 8, 10 and Fig. 11 show that the actual changes of water level of Xingshikou forebay and Mayu forebay are 0.303 m and − 0.201 m respectively. The water level errors of Xingshikou forebay and Mayu forebay are 0.003 m and − 0.001 m respectively. The error rates are reduced from 21 to 1% and from 78.5 to 0.5%, respectively. The water level error of each forebay decreased greatly, by 95.2% and 99.36% respectively.

The simulation experiment results show that the output value of the decoupling controller with error compensation method can be kept very close to the expected value, which significantly improves the control accuracy of the decoupling controller on the water level change rate of each forebay, and effectively reduces the influence of project parameter error such as unit characteristic error and forebay area error.

## Conclusion

This paper presents a cooperative control method for series multivariable coupled system. Firstly, a decoupling controller with double feedforward structure is designed. Based on the interstage feedback strategy of the control loop, the decoupling of series multivariable system is realized successfully. On this basis, the error analysis of the parameters of the decoupling controller is carried out, and the differential leading PI error compensation method is introduced, and the stability of the control system is analyzed. Secondly, the parameters of the control system are analyzed, which is combined with the physical meaning of each parameter in Beijing East-to-West Water Transfer Project, and provides an important basis for the parameter setting of the subsequent simulation experiment. Thirdly, the decoupling controller is applied to the simulation experiment of East-to-West Water Transfer Project to verify the effectiveness of the decoupling controller. The decoupling controller can eliminate the coupling relationship between each forebay and make each forebay independent of each other, and can control any one or more forebays flexibly. Finally, the decoupling controller with error compensation is applied to the simulation experiment of East-to-West Water Transfer Project, and it is verified that the error compensation method can suppress the influence of project parameter error such as the characteristic error of pumping station units and the error of the forebay area on the control accuracy of the water level change rate of each forebay, which makes the decoupling controller have better control performance.

## Data Availability

Data are fully available through the corresponding author upon reasonable request.
